# Health-related quality of life in older survivors of melanoma: a SEER-MHOS study

**DOI:** 10.1007/s11136-026-04259-z

**Published:** 2026-06-05

**Authors:** Divya M. Shan, Lindsay Irwin, Benjamin T. Allaire, Mark C. Mochel, Molly E. Maher, Roxanne E. Jensen

**Affiliations:** 1 Outcomes Research Branch, Healthcare Delivery Research Program, National Cancer Institute, Rockville, USA; 2 Department of Pathology and Dermatology, Virginia Commonwealth University School of Medicine, Richmond, USA; 3 RTI International, Research Triangle Park, USA

**Keywords:** Melanoma, Quality of life, Cancer disparities, Health outcomes

## Abstract

**Purpose:**

Melanoma, which accounts for only 1% of skin cancers, causes the majority of skin cancer-related deaths and morbidity. Health-related quality of life (HRQOL) outcomes in melanoma survivors have not been well-described. Given increasing melanoma survivorship in recent years, there is a need to describe and quantify HRQOL outcomes in a U.S. population-based sample of melanoma cancer survivors. This study aims to characterize HRQOL in melanoma survivors based on stage of diagnosis, demographic factors, and clinical characteristics.

**Materials and methods:**

This cross-sectional cohort study used data from the Surveillance, Epidemiology, and End Results Medicare Health Outcomes Survey (SEER-MHOS) to identify 2955 Medicare beneficiaries diagnosed with melanoma between 1998 and 2019. HRQOL was assessed using physical (PCS) and mental (MCS) component summary scores from the Veterans Rand (VR-12), along with CDC Healthy Days. We created matched non-cancer comparison groups using 1:1 propensity score matching based on covariates including sex, age, race, education, marital status, BMI, region, and medical comorbidities.

**Results:**

Less than 10% of the study population had advanced melanoma (*N* = 253). Advanced melanoma survivors were more likely to be non-White (5.9 vs. 3.4%; *p* < 0.04) and to lack a high school diploma (11.7 vs. 7.7%; *p* < 0.05) compared to those with localized melanoma. They also reported lower mean PCS (38.3 vs. 41.6; *p* < 0.05) and MCS (51.9 vs. 54.0; *p* < 0.05) scores compared to localized melanoma survivors, and lower mean PCS scores compared to the matched non-cancer group (38.3 vs. 42.0; *p* < 0.05). Furthermore, advanced melanoma survivors reported more physically unhealthy (8.3 vs. 4.8; *p* < 0.05) and activity limitation days (12.5 vs. 8.4; *p* < 0.05) compared to the matched non-cancer group.

**Conclusion:**

Individuals with advanced melanoma experience poorer physical and mental health than those with localized melanoma. The difference in physical health remains significant and is clinically meaningful when comparing advanced melanoma survivors to matched non-cancer individuals. These findings underscore the value of early melanoma detection and targeted psychosocial interventions for cancer survivors.

**Supplementary Information:**

The online version contains supplementary material available at 10.1007/s11136-026-04259-z.

## Abstract (plain language summary)

Although melanoma accounts for only 1% of skin cancers, it causes most skin cancer deaths and serious health complications. As melanoma survival rates improve, researchers recognize a critical gap: we do not know enough about how the disease affects survivors’ quality of life. The key question addressed by this research is whether melanoma, particularly advanced melanoma, meaningfully reduces survivors’ physical and mental health compared to both those with early-stage disease and people without cancer. We examined data from over 2300 Medicare beneficiaries diagnosed with melanoma between 1998 and 2019, using standard health assessment tools to measure quality of life. We compared melanoma survivors to similar individuals without cancer based on age, sex, race, education, and other key factors.

Survivors with advanced melanoma experienced significantly worse physical and mental health than those with localized melanoma. When compared to people without cancer, advanced melanoma survivors reported substantially lower physical health scores and approximately 3.5 additional days per month feeling physically unwell, plus over 4 additional days with activity limitations preventing normal tasks.

These results demonstrate that advanced melanoma has a clinically meaningful impact on survivors’ daily functioning and wellbeing. The findings support two important conclusions: early detection through regular skin screening is crucial to prevent advanced disease and its serious health consequences, and cancer survivors need targeted mental health support and counseling programs to manage the physical and emotional challenges following their diagnosis.

**Supplementary Information:**

The online version contains supplementary material available at 10.1007/s11136-026-04259-z.

## Introduction

Melanoma represents 5% of all new cancer cases in 2024 in the United States and causes more deaths than any other skin cancer [1]. Ultraviolet radiation exposure is a significant risk factor for melanoma, and prognosis varies with the cancer stage [2]. Various clinical and pathologic parameters, including depth of melanoma invasion and mitotic rate, are associated with survival [3, 4]. While mortality and recurrence are essential clinical endpoints to consider for melanoma survivors, they do not fully capture the spectrum of melanoma’s burden [5–7]. Knowledge about the long-term impact of melanoma and its treatment on health-related quality of life (HRQOL) outcomes is limited [8–11]. HRQOL-focused studies of melanoma cancer survivors are small, international, or evaluated as a secondary clinical trial endpoint [9, 12, 13]. 

HRQOL studies help characterize deficiencies in care and unmet psychosocial needs in vulnerable patient populations. The Surveillance, Epidemiology, and End Results Medical Health Outcomes Survey (SEER-MHOS) data resource has been used to study and describe the quality of life with other cancers such as bladder cancer, pancreatic ductal adenocarcinoma, and hepatocellular carcinoma, but no SEER-MHOS study to date has looked at melanoma [14–16]. There is a pressing need to describe and quantify HRQOL outcomes in a US population-based sample of melanoma cancer survivors, particularly with melanoma survivorship increasing significantly in recent years due to novel therapies [17]. In this study, the SEER-MHOS data resource was used to describe HRQOL in older survivors of localized and advanced cutaneous melanoma.

## Methods

### Data source

Using the publicly available SEER-MHOS data resource, this study identified Medicare beneficiaries diagnosed with melanoma as their first (primary) cancer between 1998 and 2019. SEER-MHOS includes information about cancer diagnosis and stage from SEER and self-reported sociodemographic factors, health problems, and HRQOL from MHOS [18, 19]. Medicare is the United States federal health insurance program that provides medical coverage to people aged 65 and older, regardless of income. Medicare includes a private insurance option, known as Medicare Advantage (MA), of which MHOS is a yearly survey of 1000–1200 randomly selected MA beneficiaries from each managed care plan with a minimum of 500 enrollees in the MA program [19]. There is also a subsequent 2-year follow-up survey [19]. Institutional Review Board approval (#HM20026023) was obtained through the Virginia Commonwealth University School of Medicine.

### Study sample

**Fig. 1:**
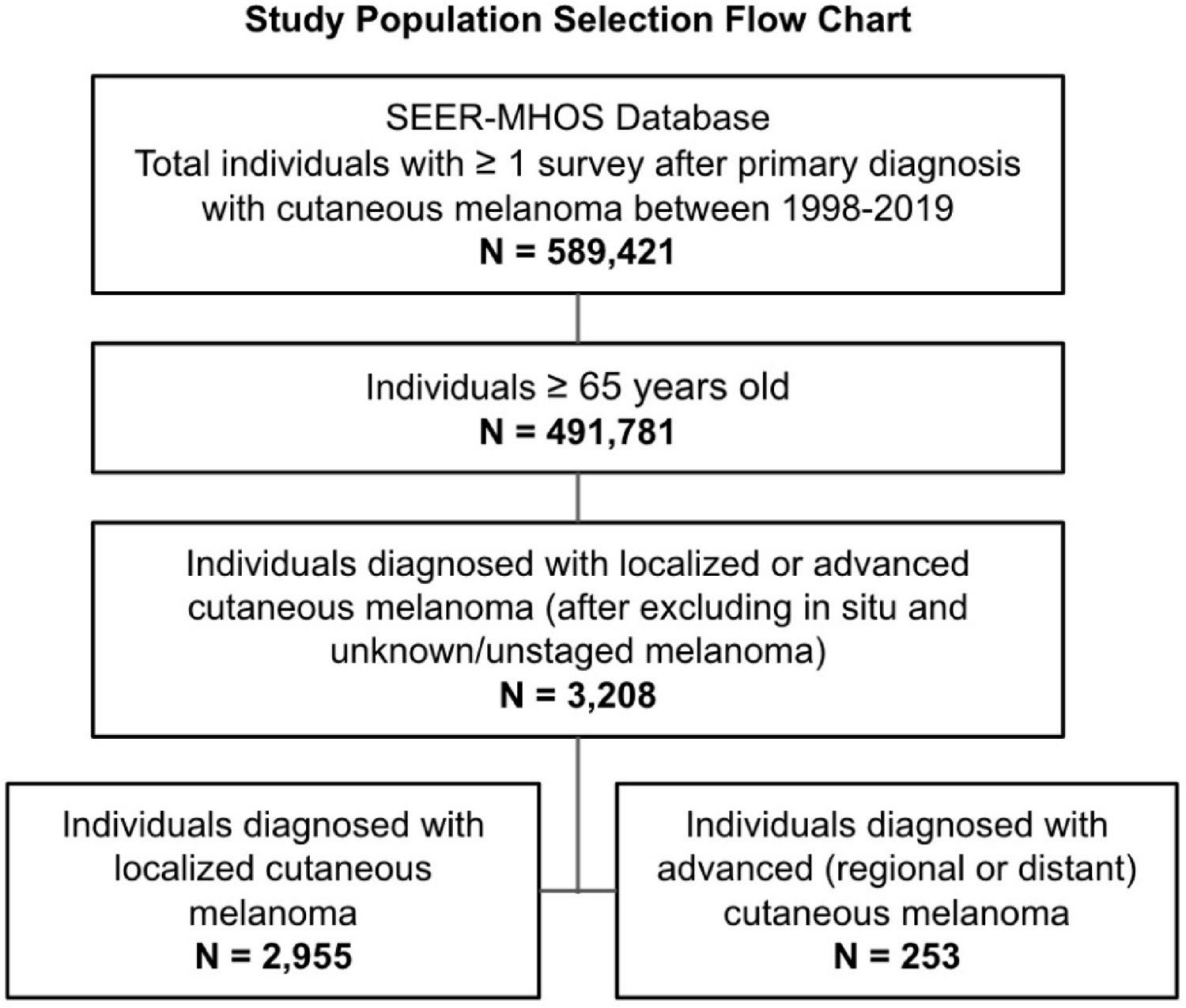
Study design. The study included individuals ≥ 65 years who completed the SEER-MHOS survey after primary diagnosis with cutaneous melanoma between 1998–2019

We initially identified 589,421 individuals with MA enrollment and primary diagnosis of cutaneous melanoma using the SEER-MHOS database between 1998 and 2019. Melanoma cases with a history of other cancers were excluded from the analysis to reduce potential confounding from other malignancies. Specifically, we selected those with an International Classification of Disease for Oncology, Third Edition (ICD-O-3) code equal to 25,010. Stage at diagnosis was classified as localized or advanced (i.e., regional or distant) cutaneous melanoma. We then excluded individuals younger than 65 years and diagnosed with in situ or unknown/unstaged melanomas. The age restriction was applied because individuals under 65 who are on Medicare typically qualify due to disability and often have more severe health conditions than those who qualify based on age. Excluding this group helps ensure that the observed outcomes are more likely attributable to cancer rather than other severe health conditions. We selected the first survey administered after diagnosis of melanoma. This resulted in a final sample of *n* = 2955 (Fig. 1).

To construct the non-cancer comparison sample, we selected the first MHOS survey completed by individuals without a history of cancer and conducted a 1:1 greedy matching on sex, age, race, education, marital status, BMI, region, and medical comorbidities for the localized and advanced melanoma groups. Then, we utilized logistic regression to predict the propensity score and performed a caliper match based on the logit of the propensity score. Matching was conducted sequentially, first matching localized cases, followed by advanced cases. To ensure temporal comparability, we exact matched on survey year so that the comparison sample was surveyed in the same year as the cancer sample. We ran balance checking analyses after each match, producing standardized differences output for all covariates, to create a balanced covariate distribution between the separately matched samples (results available in Supplemental Table 4).

### Variables

Sociodemographic and health characteristics included age at diagnosis, sex, race/ethnicity (white or non-white), highest level of education (less than high school, high school graduate, college or higher), number of comorbidities, BMI (< 30 kg/m^2^ or ≥ 30 kg/m^2^, threshold for obesity), marital status (married or not), and time since diagnosis (< 2 years, 2–5 years, 5–10 years, and > 10 years).

The primary study outcomes were health-related quality of life using physical (PCS) and mental (MCS) component summary scores and subscales from the Veterans RAND 12-item Health Survey (VR-12). The VR-12 measures HRQOL on eight domains (physical functioning, role limitations due to physical health, bodily pain, general health perceptions, emotional well-being, vitality, social functioning, and role limitations due to emotional health). A minimally important difference was considered as 2 points on PCS/MCS and 3 points on subscale scores [20]. VR-12 have been extensively validated in diverse populations, including patients with cancer, with the ability to differentiate between individuals with different health conditions, measuring eight health domains [21–25]. 

Additional outcome measures included unhealthy days and activity limitation days from the CDC Healthy Days HRQOL measures, which has also been validated in populations such as Medicare Advantage members [25]. The CDC Healthy Day measures are split into four scales representing the number of days within the last 30-day period. Scores are calculated for physically unhealthy days, mentally unhealthy days (includes stress, depression, problems with emotions), unhealthy days (respondent feeling their physical or mental health was poor), and activity limitation days (due to poor physical or mental health) [26]. 

## Statistical analysis

We calculated descriptive statistics for the study population, reporting means and standardized deviations for continuous variables and percentages for categorical variables (Table 1). All counts < 11 for categorical variables were suppressed to maintain confidentiality. Differences in between continuous and categorical variables between the localized and advanced melanoma groups were calculated using t-tests and chi-square tests, respectively.

**Table 1:** Demographic and health characteristics of melanoma survivors

	Localized melanoma (*N* = 2955)	Advanced melanoma (*N* = 253)	*p*-value for differences
Age at survey, mean (SD)	75.5 (7.0)	75.7 (6.8)	0.65
**Sex, N (%)**			
Male	1753 (59.3%)	152 (60.3%)	0.75
Female	1202 (40.7%)	100 (40.7%)	
**Race/ethnicity, N (%)**			
White	2854 (96.6%)	237 (94.0%)	0.04
Asian, Black, Hispanic, American Indian, Other*	101 (3.4%)	15 (5.9%)	
**Education, N (%)**			
Some high school or less	259 (7.7%)	396 (11.7%)	< 0.01
High school graduate or GED	1564 (46.2%)	1898 (56.1%)	
College graduate or higher	1074 (31.7%)	1091 (32.2%)	
**Comorbidity**			
Pulmonary	357 (12.1%)	39 (15.5%)	0.12
Hypertension	1906 (64.5%)	161 (63.9%)	0.85
Cardiovascular	1115 (37.7%)	91 (36.1%)	0.61
Musculoskeletal	1660 (56.2%)	127 (50.4%)	0.08
**BMI, N (%)**			
< 30 kg/m^2	2015 (58.2%)	2314 (66.9%)	0.49
≥ 30 kg/m^2	732 (21.2%)	883 (25.5%)	.
**Primary site, N (%)**			
Head/neck	689 (23.3%)	56 (22.2%)	0.69
Trunk	931 (31.5%)	59 (23.4%)	0.01
Upper extremity	846 (28.6%)	60 (23.8%)	0.10
Lower extremity	469 (15.9%)	51 (20.2%)	0.07
Multisite/other	20 (0.7%)	26 (10.3%)	< 0.01
Married, Yes, N (%)	1902 (64.4%)	146 (57.9%)	0.04
Proxy response, N (%)	175 (5.9%)	14 (5.6%)	0.81
**Time since diagnosis**			
< 2 years	880 (29.8%)	106 (42.1%)	< 0.01
2–5 years	969 (32.8%)	73 (29.0%)	
6–10 years	706 (23.9%)	55 (21.8%)	
> 10 years	400 (13.5%)	18 (7.1%)	
Ulceration, Yes (%)	69 (2.3%)	20 (7.9%)	< 0.01

*Asian or Pacific Islander, Black or African American, Hispanic, American Indian or Alaskan Native, Other or Multi-Race

**Incomplete percentages represent missing values

We examined MCS and PCS differences between the localized and advanced melanoma groups, as well as the matched non-cancer comparison groups, using t-tests (Tables 2 and 3). We implemented the same statistical tests for comparing VR-12 subscales as well as CDC Healthy Days between the localized and advanced melanoma groups, as well as the matched non-cancer comparison groups. Statistical significance was at *p* < 0.05 and determined using the general linear model (GLM) procedure. All statistical analysis for this study was performed with Stata 17 (Stata Corporation, College Station, TX).

**Table 2:** Comparison of mean reported scores from SEER-MHOS respondents with localized vs. advanced melanoma

	Localized melanoma (*N* = 2955)	Advanced melanoma (*N* = 253)	Difference (95% CI)
**VR-12 summary scores**			
PCS	41.6 (41.2, 42.1)	38.3 (36.8, 39.7)	3.4* (1.8, 4.9)
MCS	54.0 (53.6, 54.3)	51.9 (50.6, 53.2)	2.1* (0.8, 3.4)
**CDC healthy days**			
Physically unhealthy days	5.7 (5.4, 6.1)	8.3 (6.8, 9.8)	− 2.5* (− 3.9, − 1.2)
Mentally unhealthy days	3.1 (2.8, 3.4)	4.2 (2.9, 5.5)	− 1.1* (− 2.1, 0.0)
Unhealthy days	3.8 (3.5, 4.1)	5.5 (4.2, 6.9)	− 1.8* (− 2.9, − 0.6)
Activity limitation days	8.9 (8.3, 9.4)	12.5 (10.2, 14.8)	− 3.6* (− 5.6, − 1.6)

95% confidence intervals presented in parenthesis

*P < 0.05

**Table 3:** Comparison of mean reported scores from SEER-MHOS respondents with and without localized and advanced melanoma

	Localized melanoma (*N* = 2955)	Matched non-cancer group for localized melanoma (*N* = 2955)	Difference	Advanced melanoma (*N* = 253)	Matched non-cancer group for advanced melanoma (*N* = 253)	Difference
**VR-12 summary scores**						
PCS	41.6 (41.2, 42.1)	41.4 (41.0, 41.8)	0.2 (− 0.4, 0.8)	38.3 (36.8, 39.7)	42.0 (40.6, 43.5)	− 3.8* (− 5.8, − 1.7)
MCS	54.0 (53.6, 54.3)	53.3 (52.9, 53.7)	0.7 (0.2, 1.2)	51.9 (50.6, 53.2)	52.8 (51.4, 54.1)	− 0.9 (− 2.8, 0.9)
**CDC healthy days**						
Physically unhealthy days	5.7 (5.4, 6.1)	5.8 (5.4, 6.2)	− 0.1 (− 0.6, 0.5)	8.3 (6.8, 9.8)	4.8 (3.8, 5.9)	3.4* (1.6, 5.3)
Mentally unhealthy days	3.1 (2.8, 3.4)	3.4 (3.1, 3.7)	− 0.3 (− 0.7, 0.1)	4.2 (2.9, 5.5)	3.6 (2.5, 4.7)	0.6 (− 1.1, 2.3)
Unhealthy days	3.8 (3.5, 4.1)	4.2 (3.9, 4.5)	− 0.4 (− 0.9, 0.1)	5.5 (4.2, 6.9)	3.9 (2.8, 5.0)	1.7 (− 0.1, 3.4)
Activity limitation days	8.9 (8.3, 9.4)	9.2 (8.6, 9.8)	− 0.4 (-1.2, 0.4)	12.5 (10.2, 14.8)	8.4 (6.7, 10.2)	4.0* (1.2, 6.9)

95% confidence intervals presented in parenthesis. The non-cancer comparison group was matched for the following characteristics: sex, age, race, education, marital status, BMI, region, and comorbidities

*P < 0.05

## Results

### Survivor characteristics

The demographic characteristics of the final sample of 2955 melanoma survivors are presented in Table 1. In our final analytic sample (*n* = 2955), 8.6% were diagnosed with advanced melanoma (*n* = 253). The majority of the study population was white (96.6%), male (59.3%), and had at least a high school degree (77.9%). Individuals with advanced melanoma were more likely to be non-White (including Asian or Pacific Islander, Black or African American, Hispanic, or other) compared to those with localized melanoma (5.9 vs. 3.4%; *p* = 0.04). Additionally, individuals with advanced melanoma had lower educational attainment, with 11.7% lacking a high school degree, compared to 7.7% among those with localized melanoma *(p* < 0.01). Lesion location also varied significantly between the two groups; individuals with localized melanoma were more likely to have lesions on their trunk (31.5 vs. 23.4%; *p* < 0.01), whereas those with advanced melanoma had a higher prevalence of lesions on their lower extremities (20.2 vs. 15.9%; *p* = 0.07). Ulceration, an adverse prognostic finding for melanoma, was present in 7.9% of individuals with advanced melanoma, compared to 2.3% in those with localized melanoma *(p* < 0.01*).* [27]

### HRQOL in localized and advanced melanoma survivors

The comparison between localized and advanced melanoma survivors in Table 2 shows significant differences in several HRQOL measures. Advanced melanoma survivors reported lower PCS (38.3 vs. 41.6, *p* < 0.05) and MCS (51.9 vs. 54.0; *p* < 0.05) scores compared to those with localized melanoma, indicating worse physical and mental health, respectively. They also reported more physically unhealthy (8.3 vs. 5.7, *p* < 0.05), mentally unhealthy (4.2 vs. 3.1; *p* < 0.05), and activity limitation days (12.5 vs. 8.9, *p* < 0.05).

In Table 3, individuals with advanced melanoma report lower PCS scores (38.3 vs. 42.0; *p* < 0.05) compared to matched non-cancer individuals. Advanced melanoma survivors also reported more physically unhealthy (8.3 vs. 4.8, *p* < 0.05) and activity limitation days (12.5 vs. 8.4, *p* < 0.05) compared to matched non-cancer individuals. There were no significant differences in HRQOL between individuals with localized melanoma and matched non-cancer controls. Additional sub score results are available in Supplementary Table 5.

## Discussion

Advanced melanoma survivors reported poorer physical health and mental health compared to those with localized melanoma. They also demonstrated worse physical health compared to the matched non-cancer group, after adjusting for sex, age, race, education, marital status, BMI, region, and comorbidities. The difference in physical health between advanced melanoma survivors and both localized melanoma survivors and the matched non-cancer group was clinically meaningful (≥ 2 point difference in PCS), reflecting poorer self-reported performance in activities, greater pain interference, lower energy, and other negative qualities [20]. Furthermore, advanced melanoma survivors reported more physically unhealthy and activity limitation days than both localized melanoma survivors and matched non-cancer individuals.

While mental health scores differ significantly between localized and advanced melanoma survivors, these differences were not consistently clinically meaningful when compared with non-cancer controls. HRQOL among individuals with localized melanoma was comparable to that of matched non-cancer controls, suggesting that early-stage melanoma is associated with minimal functional or mental health impairment and reinforcing the benefits of early detection and treatment. This study showcases health challenges experienced by those diagnosed with advanced melanoma, highlighting of the potential value of physical therapy and rehabilitation [28]. This analysis also illustrates the potential value of targeting early detection of melanoma.

In terms of demographics, this study found that individuals with advanced melanoma were more likely to be non-White. Though cutaneous melanoma is more likely to develop in individuals with lighter skin, studies have shown that melanomas in Black patients are often found at more advanced stages with worse outcomes compared to white patients, and for stages I and III, blacks have significantly lower survival rates [29, 30]. This is supported by another study which found that African Americans and Hispanics are more likely to be diagnosed with advanced stages of melanoma due to socioeconomic barriers such as lower levels of education and melanoma awareness and knowledge [31]. Unfortunately, these populations are also more likely to have increased rates of melanoma-specific mortality [31]. 

The results of this study also demonstrate that beneficiaries with advanced melanoma were more likely to have a high school (HS) education or less. Pollitt et al. reported that HS-educated individuals were less likely to perceive themselves as at risk for developing melanoma, understand the seriousness of melanoma, and know the signs of melanoma compared to college graduates [32]. These findings highlight the gap in melanoma awareness among low socioeconomic status individuals which leads to increased diagnoses of advanced melanoma in this population.

Clinical differences in this population included the primary sites of melanoma, with individuals having localized melanoma more likely to present with lesions on the trunk, while those with advanced melanoma more often had lesions on the lower extremities. These findings align with several studies that have shown gender differences in malignant melanoma occurrence, with men having more lesions on their torsos and women having more lesions in the lower extremities [33, 34]. Additionally, individuals with advanced melanoma exhibited higher rates of ulceration, which aligns with the understanding that ulceration is a prognostic factor for melanoma [35]. Recognizing these patterns can aid in early detection and tailored management strategies, ultimately improving patient outcomes.

The strengths of this study include the application of well-validated HRQOL measures and clinical cancer registry data. While studies of this nature are typically performed in clinical settings, the SEER-MHOS dataset uses a community-based US sample and captures patients from a wide and diverse group of geographic areas, followed throughout a long period of survivorship. The matched non-cancer comparison groups accounted for numerous covariates such as age, education, sex, and marital status, which have been linked to HRQOL in prior studies, which further strengthens our study results [36]. 

Some weaknesses include limited generalizability of this study beyond Medicare Advantage beneficiaries and adults under the age of 65 years. These findings may not apply to younger melanoma survivors or individuals with different insurance coverage (i.e., fee-for-service Medicare or uninsured populations). However, since the average age at the time of diagnosis of melanoma is 65, this correlates with the age range of our study sample. Saginala et al [37] The smaller sample size of the advanced melanoma group (*n* = 253) compared to the localized melanoma group (*n* = 2955) might have impacted the findings, with the former group being underpowered to detect differences. Our results may contain survivorship bias, especially among individuals with advanced melanoma, because we rely on a single post-diagnosis survey. Individuals with the most aggressive disease or greatest HRQOL impairment may be underrepresented due to early mortality or inability to complete MHOS surveys. Furthermore, our categorization of advanced melanoma included both regional and distant disease, which encompass a broad spectrum of clinical presentations. This grouping was necessary due to sample size limitations but may obscure clinical differences within this population. Without stratification by metastatic site and burden or treatment status, the observed HRQOL differences may reflect averages across clinically heterogenous subgroups.

Lastly, the data presented here was pooled over a 20 + year time frame which means that we may not have captured variations in treatments and survival durations over time. Since 2011 was a landmark year for melanoma therapy with the FDA approval of two agents that improved survival, anti-CTLA4 antibody ipilimumab and BRAF inhibitor vemurafenib, we performed a sensitivity analysis comparing MCS and PCS values in melanoma survivors before and after 2011 [38]. There was no significant difference found in MCS and PCS scores between the two periods, though these results should be interpreted carefully. The analysis did not distinguish between patients receiving different forms of therapy (immunotherapy, targeted therapy, chemotherapy) and is underpowered given the sample size of our study. Other studies have shown that newer targeted therapies have improved symptoms, functioning, and HRQOL for individuals with stage III and IV cutaneous melanoma [39]. 

Due to sample size limitations, multiple racial groups were combined into an “Other” category which may obscure important differences across populations. Larger samples and increased data from ethnic minorities and medically underserved populations may provide additional insight into the impacts of race and ethnicity on HRQOL. Future research could also investigate how HRQOL is impacted by melanoma recurrence, unresectable local disease, and different sites of metastasis (e.g., lung, liver, brain, etc.) to further identify subgroups of individuals with advanced melanoma who are at a higher risk for poor quality of life [40]. 

## Conclusion

Melanoma at advanced stages negatively impacts HRQOL, both in terms of physical and mental health compared to localized stages. Additionally, individuals with advanced melanoma report poorer physical health than those in the matched non-cancer group. This study, utilizing a population-based dataset, contributes to the literature on HRQOL with a specific focus on melanoma survivors. Given the high incidence of skin cancer and melanoma in the general population, these findings highlight the critical importance of early detection. Future research should explore early diagnosis and psychosocial interventions to enhance the HRQOL of melanoma survivors.

## Supplementary Information

Below is the link to the electronic supplementary material.

Supplementary file 1

## Data Availability

The data that support the findings of this study are available from the National Cancer Institute’s Surveillance, Epidemiology, and End Results–Medicare Health Outcomes Survey (SEER-MHOS) linked data resource. Restrictions apply to the availability of these data, which were used under a Data Use Agreement for this study. The SEER-MHOS data are not public use files; investigators must submit an application and receive approval from the National Cancer Institute to access the data. Information on obtaining SEER-MHOS data is available at https://healthcaredelivery.cancer.gov/seer-mhos/obtain/.
