# Supplementary material for: Health-related quality of life in older survivors of melanoma: a SEER-MHOS study
Source: Qual Life Res. 2026 Jun 5;35(7):162. doi: 10.1007/s11136-026-04259-z (PMC13241430; doi:10.1007/s11136-026-04259-z)
Supplement: Supplementary file 1 [file 11136_2026_4259_MOESM1_ESM.docx]

**Supplemental**

### Table 4. Comparison group balance assessment from SEER-MHOS respondents with and without localized and advanced melanoma

| **Matching**  **Variable** | **Localized Melanoma (N=2955)** | **Matched Non-Cancer Group for Localized Melanoma (N=2955)** | **Standard Bias** | **Advanced Melanoma (N=253)** | **Matched Non-Cancer Group for Advanced Melanoma (N=253)** | **Standard Bias** |
| --- | --- | --- | --- | --- | --- | --- |
| **Male** | 59.3% | 54.5% | 9.8 | 60.5% | 62.8% | -4.8 |
| **Age at Survey** |  |  |  |  |  |  |
| 65-69 | 24.0% | 25.8% | -4.1 | 23.3% | 23.7% | -0.9 |
| 70-74 | 26.6% | 28.0% | -3.1 | 21.7% | 20.6% | 2.7 |
| 75-79 | 20.9% | 19.6% | 3.4 | 24.5% | 24.9% | -1.0 |
| 80-84 | 16.1% | 15.0% | 3.2 | 18.6% | 16.6% | 5.5 |
| 85+ | 12.4% | 11.6% | 2.4 | 11.9% | 14.2% | -7.6 |
| **Race** |  |  |  |  |  |  |
| White | 96.6% | 96.2% | 1.0 | 93.7% | 92.1% | 4.2 |
| Other | 0.3% | 0.3% | 0.5 | 0.0% | 0.0% | 0.0 |
| **Education** |  |  |  |  |  |  |
| Some high school or less | 8.8% | 10.9% | -5.8 | 13.8% | 15.8% | -5.0 |
| High school graduate or GED | 52.9% | 54.2% | -2.6 | 58.5% | 58.1% | 0.8 |
| College graduate or higher | 36.3% | 32.6% | 8.6 | 26.5% | 24.5% | 4.7 |
| **Comorbidity** |  |  |  |  |  |  |
| Pulmonary | 12.1% | 13.2% | -3.3 | 15.4% | 16.2% | -2.2 |
| Hypertension | 64.5% | 63.2% | 2.8 | 64.0% | 55.7% | 17.4 |
| Cardiovascular | 37.7% | 37.8% | -0.1 | 36.4% | 34.0% | 5.0 |
| Musculoskeletal | 56.2% | 57.2% | -2.1 | 50.6% | 45.5% | 10.4 |
| **BMI** |  |  |  |  |  |  |
| < 30 kg/m^2 | 68.2% | 67.2% | 2.0 | 64.4% | 65.2% | -1.6 |
| ≥ 30 kg/m^2 | 24.8% | 25.1% | -0.8 | 29.2% | 26.5% | 6.2 |
| **Married** | 64.4% | 63.0% | 2.7 | 58.1% | 52.2% | 11.9 |
| **Proxy** | 5.9% | 6.5% | -1.8 | 5.5% | 7.1% | -5.4 |
| **Region** |  |  |  |  |  |  |
| NE | 26.5% | 29.3% | -6.1 | 32.8% | 36.0% | -6.6 |
| MW | 5.9% | 6.7% | -3.5 | 5.1% | 4.3% | 3.5 |
| South | 21.6% | 20.0% | 4.1 | 22.5% | 20.6% | 4.9 |
| West | 46.1% | 44.0% | 4.1 | 39.3% | 38.9% | 0.8 |
| **Survey Year** | 2013 | 2013 | 0.0 | 2013 | 2013 | 0.0 |

### Table 5. Comparison of mean reported VR-12 subscale scores from SEER-MHOS respondents with and without localized and advanced melanoma

|  | **Localized Melanoma (N=2955)** | **Matched Non-Cancer Group for Localized Melanoma (N=2955)** | **Difference**  **(Confidence Interval)** | **Advanced Melanoma (N=253)** | **Matched Non-Cancer Group for Advanced Melanoma (N=253)** | **Difference**  **(Confidence Interval)** |
| --- | --- | --- | --- | --- | --- | --- |
| **VR-12 Subscale Scores** |  |  |  |  |  |  |
| Physical Functioning (PF) | 65.5  (64.4, 66.6) | 64.6  (63.5, 65.7) | 1.0 (-0.6, 2.5) | 57.8  (53.9, 61.6) | 66.3  (62.6, 70.0) | -8.5  (-13.8, -3.3) |
| Role physical (RP) | 59.6  (58.1, 61.1) | 58.7  (57.1, 60.2) | 0.9 (-1.2, 3.1) | 46.7  (41.4, 52.1) | 60.2  (55.0, 65.4) | -13.4  (-20.9, -6.0) |
| Bodily pain (BP) | 63.3  (62.3, 64.2) | 62.1  (61.1, 63.1) | 1.2 (-0.2, 2.5) | 59.8  (56.6, 63.0) | 63.2  (59.9, 66.4) | -3.4  (-7.9, 1.1) |
| General health (GH) | 66.7  (65.8, 67.5) | 66.7  (65.9, 67.6) | -0.1 (-1.2, 1.1) | 58.5  (55.5, 61.4) | 66.0  (63.2, 68.7) | -7.5  (-11.5, -3.5) |
| Mental health (MH) | 79.3 (78.6, 79.9) | 78.0  (77.3, 78.7) | 1.2  (0.2, 2.2) | 75.7  (73.2, 78.1) | 78.4  (75.9, 80.8) | -2.7  (-6.1, 0.7) |
| Role emotional (RE) | 82.9  (81.6, 84.1) | 80.6  (79.3, 81.8) | 2.3  (0.6, 4.1) | 74.0  (69.3, 78.8) | 78.5  (74.0, 83.0) | -4.5  (-11.0, 2.0) |
| Social functioning (SF) | 80.5  (79.5, 81.4) | 79.2  (78.2, 80.2) | 1.3  (-0.1, 2.7) | 73.9  (70.4, 77.4) | 78.9  (75.5, 82.3) | -5.0  (-9.8, -0.2) |
| Vitality (VT) | 58.7  (57.9, 59.5) | 58.8  (58.0, 59.7) | -0.1  (-1.3, 1.1) | 54.5  (51.6, 57.4) | 58.1  (55.2, 61.1) | -3.6  (-7.7, 0.5) |

Note: 95% confidence intervals presented in parenthesis. The non-cancer comparison group was matched for the following characteristics: sex, age, race, education, marital status, BMI, region, and comorbidities.

*P<0.05
